# What explains the gender differences in the adoption of multiple maize varieties? Empirical evidence from Uganda and Tanzania

**DOI:** 10.1016/j.wdp.2020.100206

**Published:** 2020-06

**Authors:** Hailemariam Teklewold, Rahma I. Adam, Paswel Marenya

**Affiliations:** a Environment & Climate Research Center (ECRC)/Policy Studies Institute (PSI), P.O. Box 2479, Addis Ababa, Ethiopia; b International Maize and Wheat Improvement Center (CIMMYT), c/o The World Agroforestry Center, ICRAF House, United Nations Avenue, Gigiri, P.O. Box 1041, Nairobi 0062, Kenya

**Keywords:** Adoption, Gender, Multiple maize varieties, Tanzania, Uganda

## Abstract

Understanding the process that underpins the effective and equitable adoption of modern crop varieties remains an imperative for agricultural development in Africa. This study examines gender differences in adoption rates and determinants of the decision to adopt drought-tolerant (DT) and non-drought-tolerant (ND) maize varieties, based on analysis of maize production data from Tanzania and Uganda. Applying a switching regression with multinomial logit models, we exploit plot level adoption decisions by women and men individually or jointly with in the household, controlling for gender dimension of resource ownership along with other covariates. We find gender differences in the adoption rates for both DT and ND. The results suggest substitution effects noticeable in the decision to use one maize variety rather than another. As the size of the area about which both spouses are jointly owned increases, so does the likelihood that they will adopt DT. Furthermore, DT adoption is also more likely if farmers have experienced frequent climate shocks and dry spells in the past growing seasons. The differences in adoption behavior between men and women jointly or individually are attributable to a combination of the levels and returns of physical- and social-capital factors, as well as to structural issues. One important policy implication of the results is the need for targeted and disaggregated strategies for scaling modern maize varieties, instead of one-size-fits-all approaches.

## Highlights

• We examine gender gap and factors explaining adoption of a mix of maize varieties.
• Farmers make adoption decisions jointly and substituting one type over the other.
• Heterogeneous effects of intra household resource ownership on adoption.
• Farmers adopt drought tolerant varieties if they experience climate shocks.
• The results indicated return gaps and composition effects on adoption status.

## 1 Introduction

Maize production in sub-Saharan Africa (SSA) is plagued with a myriad of challenges that include pests, diseases, weeds, poor agronomic practices and adverse climate change. Faced with different types of shocks that result in reduced agricultural yields and increased yield variability, risk-averse farmers usually apply a variety of ex-ante strategies; this results in less production volatility, but often in a lower average production (Bryan, Ringler, Okoba, Koo, Herrero, & Silvestri, 2011). In maize cultivation, these strategies may include the adoption of drought-tolerant (DT) varieties, or a combination of drought-tolerant and non-drought-tolerant (ND) varieties. The latter implies intra-crop diversification by simultaneously planting multiple maize varieties with different response characteristics to climate- or environmental stresses (Bellon and Berthaud, 2006, Teklewold et al., 2013). While the DT varieties are adopted as adaptation strategies, the ND varieties are cultivated for traits other than drought tolerance, such as market-related qualities and consumer preferences (Fisher et al., 2015). This is especially true if the DT varieties lack specific traits sought by farmers and are planted for the purpose of resisting drought.

Conceivably, farm households can adopt different farm technologies in order to exploit the potential advantage of these as complements, substitutes, or supplements that deal with their overlapping constraints (Teklewold et al., 2013, Kassie et al., 2015). However, many of the previous studies has been to deal with a single crop variety, or with a set of varieties considered as a single unit or independently. Hence, empirical studies that acknowledge the potential cross correlation across the adoption of DT and ND varieties, and examine decisions to adopt different type of varieties, fill a knowledge gap in the adoption literature. This study provides evidence of gender differences on the decision and determinants of the adoption of different maize varieties in two African countries: Uganda and Tanzania.

Previous studies has identified that, among other factors, gender inequalities in the adoption of technologies have long been a salient feature in most developing countries (Diiro et al., 2018, Seymour et al., 2016; Wambugu et al., 2018; Fisher & Carr, 2015; Kassie et al., 2014, Haider et al., 2018), and that improving women’s access to resources can help to reduce the equality gap (Fisher & Kandiwa, 2014). Women have less access than men to information, financial services and other resources needed for technology adoption (Diiro et al., 2018). The empirical literature on gender differences in risk-aversion behavior indicates that women are less willing to take risks than men (Dohmen, Falk, Huffman, Sunde, Schupp, & Wagner, 2005). This implies that male and female farmers often prefer different crops or crop varieties; while men often prefer high-yielding varieties that they value because they can sell the surplus produce, women’s risk-aversion behavior tends to influence their priorities towards food security and risk-reducing options (Beuchelt & Badstue, 2013).

Given women's important roles and responsibilities in the agricultural production, it is imperative to understand the gender-maize technology adoption gaps and the potential factors that inhibit women in raising the adoption level. Most of the previous studies on gender and adoption of improved maize varieties have used the sex of the household head or farmer to represent gender. For instance, Ricker-Gilbert and Jones, 2015, Bezu et al., 2014 found no significant difference in the adoption of maize varieties between male and female headed households. Others have found male-headed households to be more likely to adopt improved maize compared to female-headed households (Wambugu et al., 2018, Lunduka et al., 2017, Wang et al., 2017). Fisher and Kandiwa (2014), found that female-headed households were 11 percent less likely to grow modern maize varieties as compared to male-headed households. But because the gender of the household head is not always a good indicator of women decision making for the entire farm, the result from these studies provide only limited information about a small segment of women producers in female headed households and ignores the majority of women farmers in male headed households who are the actual decision makers on the farm plots they manage (Ndiritu, Kassie, & Shiferaw, 2014).

Given the fact that decisions about technology adoption usually involve contributions from the different household members individually or jointly (Marenya, Kassie, Jaleta, & Rahut, 2016; Fisher & Carr, 2015), the gender research requires the selection of the appropriate unit of analysis. Using the parcel-level analysis, Haider et al., 2018, Ndiritu et al., 2014 have respectively shown large productivity and decision-making differences across parcels with in the same households depending on the sex of the farmer manager. Thus, the unitary household model, where the household head is the only decision-making unit used to explain adoption patterns, fails to take into account the role played by the different groups of household members, who may also own resources and be responsible for making decisions. Diiro et al. (2018) considered the relationship between women's empowerment and agricultural productivity in terms of a collective model of intra-household bargaining, in which households are considered as a collection of discrete individuals, each with his/her own set of preferences and economic status. Recent studies have also shown that the relative bargaining power of women and men within a household largely depends on their relative access to, control over, and utilization of resources (Wekwete, 2013, Kassie et al., 2014). However, understanding the gender role played by intra-household resource dynamics in the adoption of multiple maize varieties is a knowledge gap which this study aims to fill.

This paper examines an important research questions concerning gender and adoption of ND and DT maize varieties across farm plots in in Uganda and Tanzania by identifying plots of maize individually or jointly managed by men and women. Specifically, we apply switching regression with multinomial logit model to test for gender differential in adoption rates and determinants of the adoption of different type of maize varieties; and decompose the gender gap in the likelihood of adoption between the part accounted for by differences in the composition of resources, and the part accounted for by differences in returns to resources.

Our study contributes to the literature on technology adoption and gender in the following ways: we consider applying an empirical method that estimates the gap in adoption of the different types of improved maize varieties among gender of plot managers in which the women and men have sole or joint control over agricultural decision making. The study approach recognizes that gender gap in adoption of DT and ND varieties could be explained mainly by differences in socio-economic and environmental characteristics, or whether they are mainly due to behavioral differences. Our study also contributes to previous studies on the determinants of household technology adoption by choosing the collective household model that explicitly allows for the heterogeneity of households; when we examine the adoption of different types of maize varieties by gender of different plot managers who take agricultural decisions, we take into account differences in economic position as a proxy for bargaining power between individuals in the household, usually husband and wife, important for understanding intra-household resource dynamics.

The paper proceeds as follows: the next section highlight pertinent features of the study countries. The presentation of the empirical strategy follows. Results are discussed in section 4. The final section concludes.

## 2 Country context: Uganda and Tanzania

Maize is a major staple and strategic food security crop in both Uganda and Tanzania. Nationwide, small-scale farmers dominate maize production, and account for roughly 85% of the total maize production. Despite the advantages that both countries are endowed with considerable fertile arable land and diverse climatic zones, agricultural productivity remains low in both Uganda and Tanzania due to low use of improved seed, fertilizer and agro-chemicals (World Bank, 2011). Moreover, the low productivity is also attributed to the poor adaptation of most maize varieties to multiple stresses that included adverse climatic conditions such as high temperatures, drought, as well as other biotic stresses such as pests and diseases. It is against this background, that the Drought-Tolerant Maize for Africa Seed Scaling (DTMASS) project was launched in 2014 with the aim of significantly enhancing the adoption of new, climate-resilient maize seed in the targeted countries including Tanzania and Uganda. The project goal is to increase adoption of these varieties among these households to improve food security and increased incomes for smallholder farmers.

The gender-technology adoption gap is still an on-going debate. There is a paucity of research that systematically analyzes at whether gender disaggregated plots managed individually or jointly by men, women or both together fundamentally differ in the adoption of farm technologies and whether these observed difference is due to the differential access to resources among gender plot managers or behavioral differences. Uganda and Tanzania are known with the culture of gender disaggregated plot management by men and women within the same household. In terms of land ownership, the Tanzania’s 1999 Land Law, for example, provides co-ownership of land to both spouses and does not allow village councils from discriminating against women (Jacobs, 2002). Uganda’s 1998 Land Action and Condominium Law recognize women’s equal right to buy and own land and housing (Grown & Gupta, 2005). Even though the legal land status for women has improved in both countries since the legislation of land laws in both countries, Tanzanian and Ugandan women still face discrimination which limits women’s economic autonomy in terms of access to agricultural resources, land, benefiting from the revenue of the sales of crops, among other limitations (Jacobs, 2002, Grown and Gupta, 2005). This situation provides an important research avenue to analyze the gender-related differences in the adoption of different type of maize varieties that can help better identify the different constraints faced by men and women and how this will improve the improved maize adoption patterns and production potentials.

## 3 Methodology

### 3.1 Study areas and sampling

**Fig. 1:**
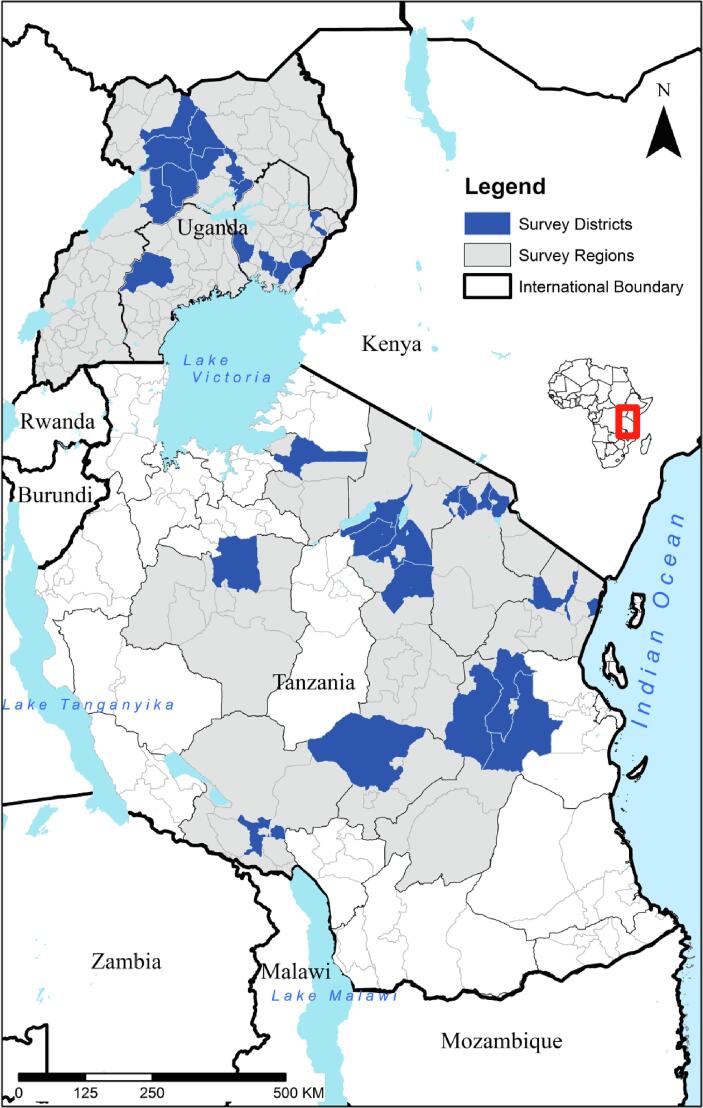
Spatial distribution of the study sites in Uganda and Tanzania.

The data for this study are drawn from a survey of rural households in Uganda and Tanzania which was collected in October and November 2015 by the International Maize and Wheat Improvement Center (CIMMYT) in partnership with the respective country’s Agricultural Research Institute. For the sampling frame, a multi-stage sampling technique was employed to select regions, villages and households, combining purposive and random sampling. A probability-proportional-to-size sample design was then used to select villages from the randomly selected regions. The third stage involved a random sampling of households within each village. The data covered 1000 households farming 2059 maize plots selected from 42 villages across 14 districts and 4 regions in Uganda, and 1020 households farming 1458 maize plots of land selected from 39 villages across 17 districts and 10 regions in Tanzania (Fig. 1).

### 3.2 Data and descriptive statistics: dependent and explanatory variables

A structured questionnaire was prepared, and the sampled respondents in both countries were interviewed using trained and experienced enumerators who were knowledgeable about the farming systems and local language of the respective countries. In most cases, the interview was conducted jointly with the household head as well as with the spouse to increase the accuracy of the data. Households were asked to provide detailed descriptions of their households and farms, including information about the following: market access; household composition; education; ownership of assets; herd size; participation in credit markets; membership of formal and informal organizations; shocks experienced; participation in extension services; crop production; land tenure; adoption of different types of improved maize varieties; a wide range of plot-specific attributes such as soil fertility, depth, slope, farm size, and area allocated to different maize varieties; and intra-household decisions concerning maize production.

#### 3.2.1 Dependent variable

The dependent variable in this study is the probability of the adoption of DT and ND maize varieties. Table 1 shows the sample distribution of adoption of DT and ND varieties gender of plot managers^1^. Without implying any causal relationship, the descriptive statistics show that adoption status is patterned by country and by gender of the plot manager. About 51% and 12% of plots managed by men in Uganda and 77% and 9% male managed plots in Tanzania are respectively covered by ND and DT maize varieties. For female managed plots, the comparable percentages are 34% and 11% for ND and DT, respectively, in Uganda and 65% and 12% for ND and DT, respectively, in Tanzania.

**Table 1:** The probability of adoption of DT and ND varieties by gender of plot manager.

Variable	Descriptions	Uganda				Tanzania			
		ALL	Men manager	Women manager	Joint manager	ALL	Men manager	Women manager	Joint manager
LV	1 = If grow local varieties	0.448	0.374	0.554	0.392	0.163	0.140	0.224	0.165
ND	1 = If grow ND varieties	0.489	0.509	0.337	0.446	0.756	0.773	0.652	0.771
DT	1 = If grow DT varieties	0.152	0.116	0.108	0.161	0.081	0.087	0.124	0.063

#### 3.2.2 Control variables

The selection of our empirical specification draws on previous similar adoption studies (Fisher et al., 2015, Fisher and Carr, 2015, Fisher and Kandiwa, 2014, Kassie et al., 2014, Kassie et al., 2015, Khonje et al., 2015, Lunduka et al., 2017, Mathenge et al., 2014, Ndiritu et al., 2014, Teklewold et al., 2013, Tura et al., 2010, Wang et al., 2017, Wekwete, 2013, Jaleta et al., 2015, Seymour et al., 2016; Diiro et al., 2018; Wambugu et al., 2018). Accordingly, detailed household- and plot-level data for each individual farm household were collected and included in the models. These contained data disaggregated by gender, and included farm features (soil depth, slope, fertility, farm fragmentation, tenure and agricultural practices); social capital (membership of community-based institutions); access to extension services; distance to main market; self-reported experiences of climate shocks, dry spells, and associated degree of damage; physical capital (farm size, livestock, assets, off-farm participation and access to credit service); household features (family size, education, age, intra-household decision-making); and location variables (e.g. altitude).

Table 2 shows gender heterogeneity in plot management among men headed households (MHHs) and women headed households (WHHs). Household headship in our sample is self-declared. Out of the self-declared WHHs some do have a spouse and the dejure WHHs are without a co-resident spouse because they were separated, divorced, widowed, or never married. In both Uganda and Tanzania, on average, about 85% of the sample households were MHHs. Of the remaining 15% of the sample, 12% were de jure FHHs and 3% were de facto FHHs. On the other hand, out of the total maize plots considered in this study, 21% in Uganda and 14% in Tanzania have women managers who decide on choice of crop and other farming decisions on the parcel. While about 42% and 40% of maize plots in Uganda and Tanzania, respectively, are managed by men individually; 38% and 47% of maize plots respectively in Uganda and Tanzania are managed by men jointly with women. The latter result shows intra-household gender heterogeneities in making decisions on choice of maize crop varieties in Uganda and Tanzania. In both Uganda and Tanzania, women managed more than 50% of maize plots in MHHs either individually or jointly with men. The above results are indicating how studies that treat only the gender of the household head would miss such intra-household heterogeneities in farming decisions.

**Table 2:** Gender differences in plot manager among men and women headed households (MHHs and WHHs).

Household head	Uganda				Tanzania			
	Plot manager							
	Men	Women	Joint	Total	Men	Women	Joint	Total
Women	2(0.76%)	231 (87.50%)	31 (11.74%)	264 (100%)	8(3.74%)	177 (82.71%)	29 (13.55%)	214 (100%)
Men	858 (47.80%	193 (10.75%)	744 (41.45%)	1795 (100%)	570 (45.82%)	24 (1.93%)	650 (52.25%)	1244 (100%)
Total	860 (41.77%)	424 (20.59%)	775 (37.64%)	2059 (100%)	578 (39.64%)	201 (13.79%)	679 (46.57%)	1458 (100%)

The description and summary statistics of the explanatory variables discussed below are presented in Table 3, Table 4.

**Table 3:** Description and summary statistics (average) of the control variables disaggregated by plot managers.

Variable	Descriptions	Uganda				Tanzania			
		Plot manager							
		All	Men	Women	Joint	All	Men	Women	Joint
**Shock**									
Shockafect	Frequency of climate shocks over the last 10 years	3.068	3.003	3.441	2.935	2.688	2.941	2.642	2.486
Dryindex	Index for dry spells experience (1 = High)	0.431	0.431	0.475	0.405	0.320	0.346	0.337	0.294
Leadindex	Index for damage with dry spells (1 = High)	0.332	0.321	0.375	0.320	0.273	0.303	0.283	0.246
**Farm features**									
Maizarea	Maize area, acre	4.097	4.302	3.430	4.234	4.979	5.268	3.385	5.204
Doublecrop	1 = If use double cropping	0.974	0.988	0.946	0.973	0.373	0.362	0.393	0.377
Intercrop	1 = If use intercropping	0.608	0.607	0.689	0.564	0.610	0.597	0.662	0.605
Fertuse	1 = If use fertilizer	0.093	0.102	0.075	0.092	0.209	0.197	0.154	0.234
Tenure	1 = if own land	0.814	0.794	0.797	0.845	0.859	0.867	0.811	0.866
Goodfert	1 = if higher fertile land	0.589	0.620	0.481	0.613	0.497	0.509	0.493	0.487
Medumfert	1 = if medium fertile land	0.348	0.322	0.446	0.324	0.440	0.441	0.388	0.454
Flatslop	1 = if steep slope land	0.608	0.593	0.618	0.619	0.569	0.587	0.577	0.552
Medumslop	1 = if medium slope land	0.303	0.317	0.295	0.290	0.317	0.296	0.303	0.339
Menland	Share of land owned by men	0.816	0.979	0.410	0.859	0.527	0.830	0.015	0.420
Womenland	Share of land owned by women	0.136	0.007	0.555	0.046	0.120	0.003	0.751	0.032
Jointland	Share of land jointly owned by men and women	0.049	0.014	0.035	0.094	0.353	0.166	0.234	0.548
Menoutput	Share of outputs controlled by men	0.440	0.875	0.028	0.186	0.345	0.836	0.000	0.029
Womenoutput	Share of outputs controlled by women	0.202	0.021	0.902	0.017	0.136	0.002	0.970	0.004
Jointoutput	Share of outputs jointly controlled by men and women	0.358	0.104	0.070	0.797	0.519	0.163	0.030	0.966
Number of observations		2059	860	424	775	1458	578	201	679

**Table 4:** Description and summary statistics of the control variables among MHHs and WHHs.

Variable	Descriptions	Uganda			Tanzania		
		All	MHHs	WHHs	All	MHHs	WHHs
**Social capital & info**							
Agrimemb	1 = if member of agricultural group	0.144	0.149	0.106	0.115	0.125	0.056
Socalmemb	1 = if member of social group	0.774	0.760	0.864	0.441	0.431	0.500
Numbmembr	Number of groups which the household is a member	1.516	1.504	1.598	0.820	0.832	0.748
Climteinfo	1 = if obtain information on rainfall and temperature	0.734	0.737	0.716	0.645	0.665	0.528
Extension	1 = if get information from extension agents	0.101	0.101	0.098	0.154	0.160	0.117
Visual	1 = if get information from field days (demonstration)	0.109	0.108	0.114	0.191	0.197	0.159
Neighbour	1 = if get information from neighbour farmers	0.740	0.734	0.780	0.594	0.580	0.673
Media	1 = if get information from media	0.031	0.035	0.008	0.052	0.052	0.051
Market	Walking distance to the market, minutes	53.359	53.259	54.042	50.29	49.69	53.81
**Resource constraints**							
Offarm	1 = if participated in off-farm activities	0.169	0.188	0.042	0.108	0.114	0.075
Credit	1 = if borrow money for inputs	0.473	0.476	0.451	0.389	0.394	0.360
Assetindex	Index for ownership of assets, 1 = High	0.571	0.583	0.487	0.641	0.659	0.533
Tlu	Tropical Livestock Unit	2.015	2.049	1.782	4.86	5.28	2.43
Farmsize	Total farm size (in acres)	5.904	6.143	4.272	6.57	6.88	4.75
**Household features**							
Agehead	Age of the household head, years	42.117	41.193	48.398	49.144	48.08	55.32
Educhead	Education level of the head, years	6.458	6.780	4.265	6.344	6.644	4.603
Famlysiz	Family size (in adult equivalent)	6.661	6.755	6.023	6.135	6.308	5.131
Altitude	Altitude (meters above sea level)	1144	1143	1152	1382	1392	1329

##### 3.2.2.1 Farm features

Farm characteristics by plot manager are shown in Table 3. Under changing climatic conditions, smallholder farmers are often subjected to environmental shocks such as extreme weather events, untimely or uneven distribution of rainfall, and incidences of pests and diseases. As in Kassie et al., 2015, Teklewold et al., 2013, this study includes self-reported experiences of climate shocks and dry spells during the cropping seasons. The individual shock index was constructed to measure farm-specific experiences related to weather shocks in the preceding seasons, based on such questions as frequency of climate shocks over the last ten years (**Shockafect**), whether the household experienced dry spells during the last five cropping seasons (**Dryindex**); and whether there was damage caused by dry spells in the last five growing seasons (**Leadindex**). Responses to each of the last two questions (either yes or no) were coded as unfavorable or favorable rainfall outcomes. When averaged over the number of years we asked about (five years), we were able to find an index that provided a value close to one for the worst (unfavorable drought outcome) and zero for the best (favorable disturbance outcome). The effect of these shocks on the use of improved maize varieties depends on the type of variety. More than half of the study farmers in both Uganda and Tanzania reported that they had experienced climate shocks more than twice during the previous ten years. The data suggests that there was no significant difference in the incidence of climate shocks among gender of plot managers.

The data also reveals that the mean plot size allocated for maize production was about 4 acres in Uganda and 5 acres in Tanzania. In both countries, the average maize area managed by women was smaller than the maize area under men management or joint management. As a measure of security of ownership of land, we included the different types of farm tenure observed in the surveyed communities. Ownership of land implies better security of tenure, which increases the likelihood that farmers will capture the returns from their investments (Teklewold et al., 2013). About 80% of the cultivated land in both Uganda and Tanzania is owned, because the household has obtained it through customary grants (coordinated by local leaders), inherited it, received it as a gift, or purchased it with title. The remaining share of land is obtained through leasehold, rented, or borrowed. Perceived plot-specific attributes, including soil fertility^2^, and plot slope^3^ are included in the empirical model. Double cropping was more common in Uganda than in Tanzania. However, intercropping is common in both countries but more common in plots that are managed by women individually or jointly with men.

In order to examine how the divergent preferences of the household members affect technology choices, it is important to understand the intra-household dynamics. The bargaining between individuals in the household, usually husband and wife is important for understanding the relative strength of the decisions made by the different household members. As in Quisumbing and Maluccio (2003), we use the definition of bargaining power that encompasses the stock of human, natural, and physical assets that men and women in the household control. Decisions regarding what to grow could be affected by bargaining power, proxy by individual’s economic position. We therefore analyzed the relative bargaining power of men and women on decisions about adopting the different types of maize variety. The bargaining power of men and women in the household is obtained by measuring the share of farm area and farm outputs where men and women solely or jointly owned and controlled.

Within the household, parcels of land under sole or joint ownership vary across Uganda and Tanzania. In Uganda, about 82 and 14 percent of land in the household are under the sole ownership, respectively, by men and women. However, about 53 and 12 percent of lands in Tanzania are respectively owned by men and women. Joint ownership of land is higher in Tanzania than in Uganda. On the other hand, it seems that almost all men managed plots in both countries are owned by men themselves individually or jointly with women. This means that the share of land owned by women and managed by men is almost none. But while 55% of women managed plots are owned by women themselves, about 41% of the plots are owned by men. In Tanzania, out of the total women managed plots, 75% are owned by women themselves individually and 23% are owned jointly with men. In Uganda, although the legal constraints to women’s land ownership were eliminated with the country’s constitution, most rural women are still unaware of their rights (Kasente, Lockwood, Vivian, & Whitehead, 2002).

Our second measure of bargaining power is the share of farm outputs controlled by men and women solely or jointly. The data suggests differences in the control of farm produces where women control a small share of outputs (between 14 and 20%) in both countries. Men control about 35–42 percent of the farm outputs individually and 26–52 percent of the output jointly with women.

##### 3.2.2.2 Household features

Table 4 presents household socio-economic characteristics. The variables concerning resource constraints (farm size, livestock size in tropical livestock units (TLU), credit, value of assets, and participation in off-farm work) are expected to have a positive association with adoption decisions, because these variables often represent an opportunity to overcome liquidity constraints to implementing improved technologies.

We also studied the ways in which individuals related to wider social networks, and the effects of these networks on the adoption of DT and ND varieties. In developing countries such as Uganda and Tanzania, where information is scarce and markets function imperfectly, social networks are considered as a means of facilitating the exchange of information, and enable farmers to access inputs on schedule and overcome credit constraints and shocks (Bandiera & Rasul, 2006). Accordingly, we distinguished three social network- and capital variables: a household’s relationship with rural institutions in the village, defined as the total number of institutions where the household is a member; whether the household is a member of a group whose objectives are related to agricultural activities; and whether the household is a member of a social group whose objectives are finance-related issues or social activities.

We controlled for access to extension services by considering whether the farmer has had contact with an extension agent. However, access to an extension service per se may not be the only source of information that impacts technology adoption. Farmers may obtain information from various sources that differ in their way of disseminating information. We thus controlled whether the farmer had access to different sources of information, such as field days, the media, markets and other farmers.

### 3.3 Econometric estimation strategies

#### 3.3.1 Multinomial logit model with regime switching

We analyze the gender effects of adoption by examining adoption patterns for different maize varieties adopted by male and female plot managers individually or jointly in maize-based farming systems in Uganda and Tanzania. Most adoption studies treat the use of modern crop varieties as a discrete all-or-nothing adoption decision of a single variety. From a policy perspective, such kind of studies lack information on how multiple varieties can fit together into an overall production strategies. Adoption of multiple crop varieties is common in Ethiopia because climate, topography and socio-economic characteristics frequently vary substantially within farms and because farmers usually diversify crop production. For a given plot of land, a farmer is assumed to have preferences over a discrete set of alternative crop varieties – a choice problem that requires application of multinomial discrete choice models.

It is assumed that individual household members consider a number of possible maize variety options, and choose a particular variety (Local, DT or ND varieties) that maximizes the expected utility conditional on the decision.

For the $i^{\mathrm{th}}$farm households, the expected utility of the g^th^ plot manager, $U_{\mathrm{igk}}^{*}$, from adopting a given maize variety k,where k(k=1,..,K) is a latent variable determined by observed household and farm characteristics $\left(X_{\mathrm{ig}}\right)$ and unobserved characteristics $\left(\varepsilon_{\mathrm{igk}}\right)$:

(1) $$U_{\mathrm{igk}}^{*}\;=\;X_{\mathrm{ig}}\beta_{\mathrm{gk}}\;+\;\varepsilon_{\mathrm{igk}}$$

Let (I) be an index that denotes the farmers’ choice of maize variety, such that:

(2) $$I\;\;=\;\;k\;\text{iff}\;U_{\mathrm{igk}}^{*}\;>\;\max_{n\neq k}\;\left(U_{\mathrm{ign}}^{*}\right)\;\;\text{or}\;\;\eta_{\mathrm{igk}}\;<\;0f\text{or}\;\;\text{all}\;n\neq k$$

Where $\eta_{\mathrm{igk}}\;=\;\max_{n\neq k}\left(U_{\mathrm{ign}}^{*}\;-\;U_{\mathrm{igk}}^{*}\right)\;<\;0$ (Bourguignon, Fournier, & Gurgand, 2007).

Equation (2) implies that the farmer will choose a maize variety k to maximize the expected utility if it provides greater expected utility than any other maize varieties n≠k that is, if $\eta_{\mathrm{igk}}\;=\;\max_{n\neq k}\left(U_{\mathrm{ign}}^{*}\;-\;U_{\mathrm{igk}}^{*}\right)\;<\;0$.

Assuming that the errors $\left(\varepsilon_{\mathrm{igj}}\right)$ are independently and identically distributed with an extreme value distribution, the probability (P) that individual household member will choose a maize variety k can be specified by a multinomial logit model (MNL) (Train, 2003):

(3) $$P_{\mathrm{igk}}\;=\;\mathrm{Pr}\;\left(\eta_{\mathrm{igk}}<0|X_{\mathrm{ig}}\right)\;=\;\frac{\exp\;(\beta_{\mathrm{gk}}X_{\mathrm{ig}})}{\sum_{k=1}^{K}\exp\;(\beta_{\mathrm{gk}}X_{\mathrm{ig}})}.$$

In our econometric specification, the choice made by plot managers (men, women or joint plot managers) to use a modern maize variety is modeled using exogenous switching with a multinomial logit model.

The traditional empirical approach to dealing with differences in adoption among the gender of plot managers by including a gender dummy variable in the pooled regression assumes that the set of covariates has the same impact on all plot managers. This assumption of common slope coefficient for all groups implies that there is no interaction between the gender variable and other explanatory variables, indicating that gender has only an intercept effect or parallel shift effect. However, the chow test rejected the assumption of parallel shift, giving a strong indication that gender-specific coefficient estimates are likely to be more informative^4^. This also suggests that the underlying process that explains adoption differs between gender of the plot managers.

This methodology identifies the heterogeneous effects of various socioeconomic factors on differences in adoption status among gender of plot managers. It also helps to decompose the gap in adoption status into the part that is due to returns to resources, and the part that is due to underlying characteristics. In exogenous switching regression, the population is divided into three groups on the basis of the gender of the plot managers.

With the multinomial logit switching regression models, the probability of adoption of ND and DT variety is predicted from equation (3) for men (m), women (w) and joint (j) plot managers:

(4) $$P_{\mathrm{igk}}\;=\;P\left(I_{i}=k|\;\beta_{\mathrm{gk}}X_{\mathrm{ig}}\right)=\frac{\exp\;(\beta_{\mathrm{gk}}X_{\mathrm{ig}})}{\sum_{k=1}^{K}\exp\;(\beta_{\mathrm{gk}}X_{\mathrm{ig}})}.$$

for g = m, w or j and k = ND, DT; and I denotes the farmers’ adoption status of maize variety.

#### 3.3.2 Estimation of the gender adoption gap

The above framework can be used to estimate the average probabilities of the adoption of DT and ND varieties by plot managers, and the effects of gender relationships and heterogeneity. The conditional probabilities for each choice variable are computed by manipulating equation (4) in the actual and counterfactual scenarios:

(5a) $$P_{\mathrm{mmk}}=\mathrm{Pr}\left(I=k|\beta_{\mathrm{mk}}X_{m}\right)\;\;\;$$

(5b) $$P_{\mathrm{wwk}}=\mathrm{Pr}\left(I=k|\beta_{\mathrm{wk}}X_{w}\right)\;\;\;$$

(5c) $$P_{\mathrm{jjk}}=\mathrm{Pr}\left(I=k|\beta_{\mathrm{jk}}X_{j}\right)\;\;\;$$

(6a) $$P_{\mathrm{jmk}}=\mathrm{Pr}\left(I=k|\beta_{\mathrm{jk}}X_{m}\right)\;\;\;$$

(6b) $$P_{\mathrm{mjk}}=\mathrm{Pr}\left(I=k|\beta_{\mathrm{mk}}X_{j}\right)\;\;\;$$

(6c) $$P_{\mathrm{jwk}}=\mathrm{Pr}\left(I=k|\beta_{\mathrm{jk}}X_{w}\right)\;\;\;$$

(6d) $$P_{\mathrm{wjk}}=\mathrm{Pr}\left(I=k|\beta_{\mathrm{wk}}X_{j}\right)\;\;\;$$

(6e) $$P_{\mathrm{wmk}}=\mathrm{Pr}\left(I=k|\beta_{\mathrm{wk}}X_{m}\right)\;\;\;$$

(6f) $$P_{\mathrm{mwk}}=\mathrm{Pr}\left(I=k|\beta_{\mathrm{mk}}X_{w}\right)\;\;\;$$

The ‘actual’ adoption probabilities for men, women and joint plot managers’ adoption are the ones actually observed in the data (equation (5a), 5b and 5c, respectively). The ‘counterfactual’ scenarios show, for instance, what the expected adoption status of the women plot mangers would have been, if the returns to their observed characteristics had been the same as the current returns to men managers’ observed characteristics (equation (6e)) and vice versa (equation (6f)). Alternatively, this means what the adoption status of women managers would be if the responses (coefficients) to their characteristics had been the same as the current returns to men plot managers’ characteristics, and vice versa.

These predicted probabilities of adoption are used to derive estimates of the gender adoption gap and heterogeneity effects. For instance, the gender gap between women and men plot mangers in probability of adoption is the change in probability for women adoption probability ($\Delta P_{\mathrm{wmk}}$), if the women mangers had had the same characteristics as they do now, but the same returns to those characteristics as the men plot mangers have now. This is the women managers’ expected adoption status due to differences in returns which is obtained by subtracting (6f) from (5b), that is, the difference of the counterfactual expected adoption status of women managers (6f) and their actual expected adoption status (5b):

(7) $$\Delta P_{\mathrm{wmk}}=\mathrm{Pr}\left(I=k|\beta_{\mathrm{wk}}X_{w}\right)\;\;-\;\;\mathrm{Pr}\left(I=k|\beta_{\mathrm{mk}}X_{w}\right)\;\;for\;k=ND,DT$$

Equation (7) is the proportion of the adoption gap that is not explained by observable gender differences in the level of resources/characteristics. In other words, this measures adoption variation due to unobserved gender inequalities including unobserved characteristics of gender of the plot manager.

Similarly, the average adoption probability gap between men and women managers ($\Delta P_{\mathrm{mwk}}$)is given as the difference between the actual expected adoption status of men managers (5a) and their counterfactual expected adoption status (6e):

(8) $$\Delta P_{\mathrm{mwk}}=\mathrm{Pr}\left(I=k|\beta_{\mathrm{mk}}X_{m}\right)\;\;-\;\;\mathrm{Pr}\left(I=k|\beta_{\mathrm{wk}}X_{m}\right)\;\;for\;k=ND,DT$$

This is the increase/decrease of probability of adoption that men managers would get if they had had the same characteristics as they do now, but the same returns to those characteristics as the women managers have now.

On the other hand, the adoption gap due to differences in the level of observable resources/ characteristics (ΔH) is given as the difference between (equation (5a)) and (equation (6f)) when the men manager adoption function is used and (5b), (6e) when the women manager adoption function is used as illustrated in (equation (9)) and (equation (10)), respectively.

(9) $$\Delta H_{\mathrm{mwk}}=\mathrm{Pr}\left(I=k|\beta_{\mathrm{mk}}X_{m}\right)\;\;-\;\;\mathrm{Pr}\left(I=k|\beta_{\mathrm{mk}}X_{w}\right)\;\;for\;k=ND,DT$$

(10) $$\Delta H_{\mathrm{wmk}}=\mathrm{Pr}\left(I=k|\beta_{\mathrm{wk}}X_{m}\right)\;\;-\;\;\mathrm{Pr}\left(I=k|\beta_{\mathrm{wk}}X_{w}\right)\;\;for\;k=ND,DT$$

The gender heterogeneity effects show, respectively, what the predicted adoption gaps would have been if all plot managers’ observable resources/characteristics had faced the current men managers’ returns and the current women managers’ returns. In other words, ΔH reflects the adoption differences that would occur if the two groups differed only with respect to characteristics of resource use.

## 4 Results and discussion

### 4.1 Interdependence of adoption of DT and ND varieties

Table 5 shows that 73% of households in Tanzania and 42% in Uganda adopted ND varieties only, without growing any other maize varieties. However, a very small proportion of households (about 2% to 4%) adopted ND in combination with DT or local maize varieties. Similarly, the proportion of households that adopted DT only is higher than the proportion of households who adopted DT in combination with ND, or grew DT together with local varieties. On the other hand, about 18% of households in Tanzania and 44% of households in Uganda grew local varieties, and the majority of these grew local varieties in isolation. The adoption of different maize varieties in isolation, and the low rate of combined adoptions are observed.

**Table 5:** Joint and marginal probabilities of adoption of different maize varieties.

Percent adopting in	Uganda				Tanzania			
	Joint probability	Marginal probability			Joint probability	Marginal probability		
		LV	ND	DT		LV	ND	DT
LV only	0.389	0.389	0.000	0.000	0.148	0.148	0.000	0.000
ND only	0.415	0.000	0.415	0.000	0.726	0.000	0.726	0.000
DT only	0.107	0.000	0.000	0.107	0.066	0.000	0.000	0.066
LV and ND	0.043	0.043	0.043	0.000	0.027	0.027	0.027	0.000
LV and DT	0.014	0.014	0.000	0.014	0.003	0.003	0.000	0.003
ND and DT	0.029	0.000	0.029	0.029	0.028	0.000	0.028	0.028
LV, ND & DT	0.002	0.002	0.002	0.002	0.000	0.000	0.000	0.000
Total	1.000	0.448	0.489	0.152	1.000	0.179	0.783	0.097

The unconditional probability of the use of local maize varieties is 18% in Tanzania and 45% in Uganda (Table 6). The likelihood of using local varieties conditional with the adoption of DT or ND varieties decreased to less than 10%. This implies that the probability of the use of local varieties is greatly reduced when households adopted DT and ND varieties, suggesting substitutability between improved and local varieties. This substitutability is higher when farmers adopt a combination of DT and ND varieties rather than adopting each in isolation. Farmers in Tanzania may abandon local varieties when they adopt a combination of DT and ND varieties. Table 6 further shows that farmers may substitute one improved maize variety type for another type. This implies that a policy that increases the adoption of DT varieties may decrease the adoption of ND varieties and vice versa.

**Table 6:** Conditional and unconditional probabilities of different types maize varieties.

	Uganda			Tanzania		
	LV	ND	DT	LV	ND	DT
P(Y_k_)	0.448	0.489	0.152	0.179	0.783	0.097
P(Y_k_|LV = 1)	1.000	0.101	0.036	1.000	0.153	0.015
P(Y_k_|ND = 1)	0.092	1.000	0.063	0.035	1.000	0.037
P(Y_k_|DT = 1)	0.105	0.204	1.000	0.028	0.296	1.000
P(Y_k_|LV = 1 & ND = 1)	1.000	1.000	0.043	1.000	1.000	0.000
P(Y_k_|LV = 1 & DT = 1)	1.000	0.121	1.000	1.000	0.000	1.000
P(Y_k_|DT = 1 & ND = 1)	0.063	1.000	1.000	0.000	1.000	1.000

Y_k_ is a binary variable representing the adoption status with respect to maize variety k (k = Local variety (LV); drought tolerant variety (DT), Non-drought tolerant variety (ND).

### 4.2 Factors explaining adoption of DT and ND varieties

The results from fitting the multinomial logit model for Uganda and Tanzania are reported in Table 7, Table 8. Keeping the local varieties as the base category where results are compared (i.e. the estimated effects are relative to being non-adopters), the result tables show two sets of parameter estimates for each men, women and joint plot managers. The estimation results shed some lights on the difference between adoptions of ND and DT across gender of plot managers. The Wald test that all regression coefficients are jointly equal to zero is rejected– suggesting the model fits the data reasonably well.

**Table 7:** Results from the multinomial logit model for the adoption of DT and ND maize varieties in Uganda.

Variables	Men manager				Women manager				Joint manager			
	ND		DT		ND		DT		ND		DT	
	Coefficient	StandardError	Coefficient	StandardError	Coefficient	StandardError	Coefficient	StandardError	Coefficient	StandardError	Coefficient	StandardError
Shock												
Shockafect	−0.118**	0.052	0.053	0.062	−0.186***	0.061	−0.368**	0.183	−0.278***	0.072	0.003	0.070
Dryindex	−0.695	0.500	−0.297	0.665	−0.758	0.828	−0.978	1.689	1.526**	0.720	0.618	0.968
Leadindex	1.041*	0.555	0.033	0.840	1.955**	0.878	1.803	1.759	−0.384	0.765	−1.358	1.078
Social capital & info												
Agrimemb	0.451*	0.347	1.742***	0.472	0.201	0.509	0.558	0.752	1.147***	0.325	0.734*	0.425
Socalmemb	−0.031	0.269	0.981**	0.464	0.300	0.507	−0.676	0.685	0.713**	0.303	0.884**	0.398
Numbmembr	−0.163	0.117	−0.373**	0.158	−0.012	0.148	0.161	0.212	−0.238**	0.119	−0.111	0.120
Climteinfo	−0.023	0.195	0.627*	0.343	0.134	0.289	0.391	0.508	0.328	0.221	0.848**	0.361
Extension	−1.327	1.065	−0.546	1.195	2.748***	0.537	2.808***	0.665	0.984	0.632	1.162	0.992
Visual	−1.473	1.038	−1.409	1.183	1.766***	0.429	0.990	0.823	0.337	0.583	0.160	0.906
Neighbour	−2.395**	1.010	−1.979*	1.133	–	–	–	–	−1.116**	0.511	−1.429	0.892
Media	−1.618	1.133	−2.650**	1.318	–	–	–	–	1.068	0.909	0.508	1.250
Market	0.001	0.002	0.007***	0.003	–	–	–	–	−0.002	0.002	0.006**	0.003
Wealth												
Offarm	−0.979***	0.215	−0.452	0.331	−0.238	0.406	−0.128	0.569	−0.307	0.334	−0.486	0.498
Credit	0.259	0.185	0.967***	0.269	0.776***	0.300	−0.075	0.508	0.193	0.200	0.181	0.267
Assetindex	−0.182	0.379	−1.680***	0.638	−0.252	0.604	1.297	0.955	−0.002	0.429	0.553	0.580
Tlu	0.045	0.038	0.047	0.063	−0.093	0.064	−0.000	0.103	−0.007	0.037	−0.002	0.052
logFarmsize	−0.364**	0.168	−0.880***	0.300	0.261	0.320	−0.139	0.484	0.292	0.217	0.239	0.263
Farm features												
Doublecrop	0.091	0.774	−0.030	1.115	−0.011	0.538	1.362	1.253	0.149	0.572	2.466*	1.329
Intercrop	−0.426**	0.169	−0.580**	0.259	−0.095	0.299	−0.709	0.479	−0.140	0.191	−0.556**	0.257
Fertuse	0.965**	0.395	2.216***	0.456	0.285	0.546	2.272***	0.715	−0.492	0.360	0.291	0.590
Ownland	−0.300	0.222	−0.309	0.321	0.452	0.326	−0.169	0.488	0.462*	0.278	−0.333	0.355
Log(Maizarea)	0.245*	0.138	0.738***	0.264	−0.217	0.276	0.332	0.485	−0.127	0.188	0.179	0.234
Goodfert	0.651*	0.376	−0.052	0.470	0.775	0.562	0.481	0.726	0.652*	0.374	1.544**	0.778
Medumfert	0.661*	0.381	−0.374	0.491	0.876*	0.537	0.174	0.691	0.925**	0.385	1.874**	0.753
Flatslop	0.337	0.344	0.048	0.487	−0.933**	0.430	16.692***	0.911	0.232	0.355	0.033	0.487
Medumslop	−0.214	0.353	0.060	0.459	−0.790*	0.433	16.489***	0.800	0.115	0.354	0.036	0.491
Menareashar	−2.400*	1.226	11.968***	1.394	−2.408***	0.593	13.262***	0.960	1.137***	0.372	−0.484	0.437
Wmenareashar	−3.157*	1.667	12.332***	1.965	−2.125***	0.562	14.075***	1.058	0.793	0.571	−2.783**	1.204
Menoutshar	0.215	0.286	−0.631*	0.379	0.643	0.786	−13.924***	1.583	0.318	0.232	−0.186	0.376
Wmenoutshar	0.976	0.847	0.553	1.076	−0.117	0.498	1.815	1.596	−1.522**	0.684	1.485**	0.745
Household features												
Log(Agehead)	0.330	0.310	0.140	0.478	−0.559	0.550	−1.163	1.040	−0.749**	0.324	−1.013*	0.521
Educhead	0.072***	0.027	0.121***	0.042	0.088**	0.041	−0.177***	0.068	0.056*	0.032	0.011	0.044
Famlysiz	0.044	0.034	0.017	0.048	−0.027	0.041	0.291***	0.079	−0.014	0.041	0.118**	0.058
Log(Altitude)	−1.577*	0.853	−0.988	1.048	1.001	1.346	−0.387	2.350	3.263***	0.830	1.959	1.288
Constant	14.259**	6.450	−5.400	7.970	−4.421	9.566	−29.166*	17.379	–22.426***	6.179	−16.276*	9.715
Observations	860				424				775			
Wald statistics	χ^2^ (68) = 236; Prob > χ^2^ = 0.0000				χ^2^ (66) = 3585; Prob > χ^2^ = 0.0000				χ^2^ (68) = 248; Prob > χ^2^ = 0.0000			

*, ** and *** indicate statistical significance at 10%, 5% and 1% level.

**Table 8:** Results from the multinomial logit model for the adoption of DT and ND maize varieties in Tanzania.

Variables	Men manager				Women manager				Joint manager			
	ND		DT		ND		DT		ND		DT	
	Coefficient	StandardError	Coefficient	Standard Error	Coefficient	StandardError	Coefficient	StandardError	Coefficient	StandardError	Coefficient	StandardError
Shock												
Shockafect	0.259	0.145	0.272	0.194	0.352	0.301	0.329	0.376	0.030	0.105	0.046	0.183
Dryindex	−1.338	1.251	−0.298	1.756	−0.746	2.452	3.937	4.563	0.512	1.027	1.579	1.538
Leadindex	0.798	1.297	−0.670	2.069	−4.746*	2.735	−10.391**	4.780	0.446	1.085	0.134	1.608
Social capital & info												
Agrimemb	0.002	0.617	−0.010	0.904	14.732***	1.664	15.487***	2.611	0.385	0.445	−0.458	0.649
Socalmemb	−0.203	0.522	1.159	0.821	0.527	0.665	0.445	1.451	0.364	0.381	0.221	0.689
Numbmembr	0.254	0.322	−0.388	0.520	−0.232	0.287	−0.071	0.538	−0.104	0.172	0.341	0.273
Climteinfo	−0.232	0.300	1.422***	0.500	−0.527	0.553	−1.781	0.963	−0.227	0.256	0.049	0.408
Extension	2.722**	1.243	15.624***	1.523	0.541	1.518	0.795	2.174	2.263**	0.961	2.610*	1.492
Visual	2.441**	1.185	14.976***	1.452					1.568*	0.847	1.390	1.416
Neighbour	0.663	1.102	13.160***	1.387	−2.857**	1.173	−3.427**	1.733	0.862	0.815	−0.015	1.348
Media	2.318	1.602	15.754***	2.032					3.252**	1.274	1.963	1.916
Market	0.002	0.003	−0.002	0.004	0.008**	0.004	0.003	0.014	0.001	0.002	0.002	0.003
Wealth												
Offarm	0.526	0.602	0.784	0.786	2.031**	0.972	2.938**	1.423	1.347**	0.656	1.015	0.917
Credit	0.073	0.321	−0.247	0.464	−1.447***	0.486	−1.698**	0.664	0.146	0.271	0.051	0.446
Assetindex	−0.631	0.604	0.504	1.034	2.342**	1.007	1.595	1.464	1.293**	0.508	0.678	0.896
Tlu	0.006	0.024	−0.031	0.034	0.002	0.086	−0.126	0.117	0.038*	0.021	0.057**	0.026
logFarmsize	0.118	0.258	0.639**	0.313	0.000	0.755	−0.008	1.032	−0.196	0.244	0.067	0.373
Farm features												
Doublecrop	−0.470	0.313	−1.380**	0.573	−0.042	0.623	−2.805**	1.123	−0.056	0.261	−1.040*	0.544
Intercrop	−0.110	0.286	−0.184	0.424	0.960*	0.508	−1.630	1.052	0.044	0.242	0.759*	0.418
Fertuse	0.707*	0.398	−2.251**	1.106	0.847	0.915	−15.482***	1.530	0.566*	0.332	−1.548*	0.795
Ownland	0.323	0.883	2.712**	1.370	0.458	0.923	−0.213	1.730	0.199	0.338	1.422*	0.859
Log(Maizarea)	0.223	0.283	−0.656*	0.360	0.603	0.733	2.321**	1.137	0.201	0.230	−0.435	0.449
Goodfert	0.776*	0.493	1.937	1.256	−0.548	1.068	−0.679	1.445	0.758*	0.407	1.490	0.971
Medumfert	1.054**	0.494	2.393*	1.256	−0.831	1.055	−1.245	1.375	0.809**	0.405	1.233	0.935
Flatslop	−0.240	0.437	−0.067	0.659	0.636	0.988	0.809	1.492	−0.263	0.349	−1.513**	0.608
Medumslop	0.141	0.466	−0.027	0.694	1.650	1.352	2.677	1.751	0.230	0.401	−0.298	0.565
Menareashar	−0.000	0.838	−0.010	0.962	1.479	1.678	−11.056***	2.645	−0.188	0.260	−0.187	0.458
Wmenareashar	13.448***	1.201	1.457	1.290	−0.780	0.951	−0.304	1.491	0.165	0.622	−15.691***	1.157
Menoutshar	0.179	0.345	1.079*	0.558					−0.344	0.672	−0.741	1.438
Wmenoutshar	−17.293***	1.789	−13.614***	2.752	0.136	1.114	−2.801	1.769	14.781***	1.147	1.945	1.291
Household features												
Log(Agehead)	0.351	0.521	−0.488	0.678	0.845	1.135	0.624	1.879	−0.249	0.407	−1.124	0.754
Educhead	0.102*	0.057	0.038	0.099	0.072	0.110	0.040	0.195	−0.038	0.047	0.030	0.085
Famlysiz	0.055	0.058	−0.035	0.083	−0.038	0.166	−0.391*	0.226	0.057	0.058	0.039	0.083
Log(Altitude)	0.384	0.668	0.977	1.072	1.216	1.571	−7.943**	3.451	1.174**	0.527	−1.848*	1.021
Constant	−6.256	5.634	−24.489***	8.282	−10.537	13.924	60.934**	28.009	−9.445**	4.194	12.396	8.208
Observations	578				201				679			
Wald statistics	χ^2^ (68) = 1360; Prob > χ^2^ = 0.0000				χ^2^ (62) = 2698; Prob > χ^2^ = 0.0000				χ^2^ (68) = 1132; Prob > χ^2^ = 0.0000			

*, ** and *** indicate statistical significance at 10%, 5% and 1% level.

Results showed the importance of climate shocks in determining farmers’ choice of maize varieties in Uganda. Women plot managers are significantly less likely to adopt both ND and DT varieties if they frequently experienced climate shocks and dry spells during the growing season. These results reinforce the idea that women farmers, who are fundamentally more risk-averse (Doss and Morris, 2001, Dohmen et al., 2005) responded negatively to adopt technologies when they experienced shocks. On the other hand, the probability of adopting ND varieties on plots managed by men individually or jointly with women is negatively influenced by the frequency of climate shock. But the frequency of climate shocks does not statistically influence adoption of DT varieties on plots exclusively or jointly managed by men. This could be the case that DT variety is a risk-reducing option, so that frequently occurred unfavorable weather conditions don’t significantly affect its adoption. Consistent with the literature on adaptation techniques, DT varieties are thus important to ensure maize production under risk, while at the same time improving resilience to drought and dry spells (Teklewold, Mekonnen, & Kohlin, 2019). These different effects of climate shock on adoption of DT and ND varieties may show the substitution between DT and ND due to climate change.

The probability of adopting ND and DT on individual plots is influenced by the intra-household bargaining power measure by land ownership and control of output by men and women, but the effects depend on the gender of the plot managers and the type of maize varieties. In Uganda, the result indicated that if the fraction of the land owned exclusively by men or women in the household increases, the probability of adopting ND variety decreases but that of DT variety increases. The result suggests that the bargaining power of men and women may have different effect on the adoption of ND and DT varieties irrespective of the gender of the individual plot manager. However, on plots that are managed jointly by men and women, gender disaggregated ownership of land has shown asymmetric effect on the choice of ND and DT varieties. The result indicates that on jointly managed plots the adoption of ND is more likely with the sole ownership of land by men but the adoption of DT is less likely with the increase in the fraction of land owned by women. The effect of gender disaggregated ownership of land on adoption of DT and ND is also differs depending on whether the land is managed individually or jointly by men and women. This result would seem to support the collective household model: it shows that with the increase in the fraction of area owned by men, adoption of ND is less likely on individually managed plots but more likely on jointly managed plots. On the other hand, with the sole ownership of land by women, the probability of adoption of DT variety is lower on individually managed plots but this likelihood of adoption is higher on jointly managed plots.

We also found that in Uganda, the exclusive controls of farm outputs by men are negatively affecting the probability of adopting DT varieties on individually managed plots. However, the measures of women’s control over farm outputs translate into differential effects on the decision to adopt ND and DT varieties on jointly managed plots. Similarly, in Tanzania, when the fraction of farm output controlled by women increases, the probabilities of adopting ND and DT varieties are less likely on men managed plots but adoptions of ND are more likely on plots jointly managed by men and women. These results provide evidence that, within households, either women or men are more likely to adopt modern maize varieties when they have the resources to do so or that men and women jointly agree to adopt and the varieties are grown on individually or jointly managed plots as a result of patterns of resource ownership and control. This entails substantial cooperation between men and women in the households as well as the importance of women’s ownership and control of resources over technology adoption decisions (Fisher & Carr, 2015). One implication of the result is that agricultural technology adoption decisions cannot be considered as an isolated decision but as part of an overall household strategy (Zepeda & Castillo, 1997).

Moreover, the analysis further indicated the importance of access to institutional services (credit, extension), social capital, household resources, farm and household characteristics on gender disaggregated adoption of DT and ND varieties. Access to extension agents in Tanzania is more likely to increase the probability of adoption of ND and DT varieties on plots managed by men individually or jointly with women. Extension services, which mostly target male famers, statistically influence their decision to adopt modern inputs (Lambrecht, Vanlauwe, & Maertens, 2016). But women farmers are generally more deprived of access to information, which likely contributes to low technology adoption rates (Doss & Morris, 2001). However, in Uganda, contact with extension agents positively influences the adoption of DT and ND varieties in women managed plots. The results support the hypothesis that farmers with more access to climate-related information will be more likely to make on-farm changes (Wood, Jina, Jain, Kristjanson, & DeFries, 2014), which in our case means adopting DT varieties. Other sources of information such as neighbor farmers, media and market also play an important role, by helping to channel information about farming techniques and weather-related information to farmers. These results highlight the challenges facing the extension systems in the future, and suggest that policies should strengthen the ability and willingness of the different sources of information to include climate-related information in their portfolio.

The importance of the social capital network variable in explaining the decisions to adopt ND and DT varieties are also different in Uganda and Tanzania. Being a member of an agricultural group can provide an incentive for adoption, and has a positive effect on the choice of ND and DT varieties on women manager in Tanzania and on plots managed by men individually and jointly with women. In Uganda, such social networks may improve the flow of information about new opportunities and potential shocks, and can also confer other benefits such as better access to finance and inputs. They can also serve as an informal insurance mechanism in times of crisis (Quisumbing, 2003). There is increasing evidence of the positive effect of peer groups on the adoption of agricultural technologies: farmers who adopt a new technology will discuss the technology with member farmers in the group and share information with them about its use (Bandiera and Rasul, 2006, Fisher and Kandiwa, 2014). The result is consistent with the findings of Wood et al. (2014) who indicated that farmers who participated in social institutions were more likely to make changes in farming practices than those who were not members of such groups. Social capital as measured by households’ participation in a number of rural institutions affects negatively the probability of adoptions of DT and ND varieties in Uganda. This might be due to the dark side of social capital as in Di Falco and Bulte (2011) where social capital may constrained the incentives for working hard and induce inefficiency such that farmers may exert less effort to invest in technologies.

### 4.3 The returns and composition effects on adoption of DT and ND varieties

The results of the gender decomposition analysis from the switching regression model are presented in Table 9, Table 10. Discussion of these results largely emphases on women and men managers for the interest of clarity. We estimated the average adoption probabilities under actual and counterfactual conditions among gender of plot managers. The counterfactual is obtained by estimating the DT and ND adoption probabilities of one gender group with what they would have been if the plot managers had had the same characteristics as the other gender groups (columns A, B and C of Table 9, Table 10 in the respective countries). Column D, E and F present the gender adoption gap, computed as the difference between the above columns. The results of the decomposition analysis in general reveal that the overall gender difference in adoptions of DT and ND are due to returns to use resources gap and heterogeneity gap.

**Table 9:** Differences between gender of plot managers in probability of adoption of DT and ND in Uganda.

Variety	Group		Plot manager (g)			Adoption gaps between:		
			Men (m) [A]	Women (w) [B]	Joint (j) [C]	Joint manager Vs Men manager[D = C-A]	Joint manager Vs Women manager[E = C-B]	Women manager Vs Men manager [F = B-C]
ND	Adoption probability	$E(P_{\mathrm{gm}}\left|\beta_{g}X_{m})\right)$	0.508 (0.006)	0.463 (0.009)	0.512 (0.006)	0.004 (0.009)	0.049 (0.011)***	−0.045 (0.011)***
		$E(P_{\mathrm{gw}}\left|\beta_{g}X_{w})\right)$	0.547 (0.008)	0.337 (0.011)	0.159 (0.007)	−0.388 (0.011)***	−0.178 (0.014)***	−0.209 (0.014)***
		$E(P_{\mathrm{gj}}\left|\beta_{g}X_{j})\right)$	0.491 (0.007)	0.415 (0.009)	0.448 (0.007)	−0.042 (0.009)***	0.032 (0.012)**	−0.075 (0.012)***
	Heterogeity effects	$E(P_{\mathrm{gj}}\left|\beta_{g}X_{j})\right)$–$E(P_{\mathrm{gm}}\left|\beta_{g}X_{m})\right)$	−0.018 (0.009)**	−0.047 (0.014)***	−0.065 (0.009)***			
		$E(P_{\mathrm{gj}}\left|\beta_{g}X_{j})\right)$–$E(P_{\mathrm{gw}}\left|\beta_{g}X_{w})\right)$	−0.057 (0.011)***	0.079 (0.016)***	0.288 (0.011)***			
		$E(P_{\mathrm{gw}}\left|\beta_{g}X_{w})\right)$–$E(P_{\mathrm{gm}}\left|\beta_{g}X_{m})\right)$	0.038 (0.010)***	−0.126 (0.015)***	−0.353 (0.010)***			
DT	Adoption probability	$E(P_{\mathrm{gm}}\left|\beta_{g}X_{m})\right)$	0.116 (0.004)	0.010 (0.002)	0.144 (0.005)	0.027 (0.007)***	0.133 (0.005)***	−0.106 (0.005)***
		$E(P_{\mathrm{gw}}\left|\beta_{g}X_{w})\right)$	0.132 (0.007)	0.109 (0.008)	0.317 (0.014)	0.185 (0.016)***	0.209 (0.016)***	−0.024 (0.011)**
		$E(P_{\mathrm{gj}}\left|\beta_{g}X_{j})\right)$	0.133 (0.006)	0.071 (0.005)	0.162 (0.005)	0.029 (0.007)***	0.091 (0.007)***	−0.062 (0.007)***
	Heterogenity effects	$E(P_{\mathrm{gj}}\left|\beta_{g}X_{j})\right)$–$E(P_{\mathrm{gm}}\left|\beta_{g}X_{m})\right)$	0.016 (0.008)*	0.061 (0.005)***	0.018 (0.007)**			
		$E(P_{\mathrm{gj}}\left|\beta_{g}X_{j})\right)$–$E(P_{\mathrm{gw}}\left|\beta_{g}X_{w})\right)$	0.0004 (0.009)	−0.037 (0.009)***	−0.155 (0.013)***			
		$E(P_{\mathrm{gw}}\left|\beta_{g}X_{w})\right)$–$E(P_{\mathrm{gm}}\left|\beta_{g}X_{m})\right)$	0.016 (0.007)**	0.098 (0.006)***	0.173 (0.012)***			

Note: g is the sample group where g = m for men manager; g = w for women manager and g = j for joint manager; Figures in parenthesis are standard errors; *, ** and *** indicate statistical significance at 10%, 5% and 1% level.

**Table 10:** Differences between gender of plot managers in probability of adoption of DT and ND in Tanzania.

Variety	Group		Plot manager (g)			Adoption gaps between:		
			Men (m)[A]	Women (w)[B]	Joint (j)[C]	Joint managerVsMen manager[D = C-A]	Joint managerVsWomen manager[E = C-B]	Women managerVsMen manager[F = B-C]
ND	Adoption probability	$E(P_{\mathrm{gm}}\left|\beta_{g}X_{m})\right)$	0.778 (0.007)	0.856 (0.010)	0.740 (0.007)	−0.049 (0.011)***	−0.132 (0.013)***	0.082 (0.013)***
		$E(P_{\mathrm{gw}}\left|\beta_{g}X_{w})\right)$	0.124 (0.019)	0.652 (0.018)	0.496 (0.002)	0.372 (0.014)***	−0.156 (0.018)***	0.528 (0.026)***
		$E(P_{\mathrm{gj}}\left|\beta_{g}X_{j})\right)$	0.778 (0.006)	0.762 (0.012)	0.772 (0.006)	−0.007 (0.009)	0.012 (0.014)	−0.018 (0.014)*
	Heterogeity effects	$E(P_{\mathrm{gj}}\left|\beta_{g}X_{j})\right)$–$E(P_{\mathrm{gm}}\left|\beta_{g}X_{m})\right)$	0.005 (0.010)	−0.107 (0.016)***	0.032 (0.009)***			
		$E(P_{\mathrm{gj}}\left|\beta_{g}X_{j})\right)$–$E(P_{\mathrm{gw}}\left|\beta_{g}X_{w})\right)$	0.655 (0.016)***	0.108 (0.024)***	0.276 (0.011)***			
		$E(P_{\mathrm{gw}}\left|\beta_{g}X_{w})\right)$–$E(P_{\mathrm{gm}}\left|\beta_{g}X_{m})\right)$	−0.649 (0.017)***	−0.215 (0.021)***	−0.244 (0.013)***			
DT	Adoption probability	$E(P_{\mathrm{gm}}\left|\beta_{g}X_{m})\right)$	0.086 (0.005)	0.056 (0.008)	0.055 (0.004)	−0.032 (0.007)**	0.002 (0.009)	−0.030 (0.009)***
		$E(P_{\mathrm{gw}}\left|\beta_{g}X_{w})\right)$	0.001 (0.001)	0.124 (0.013)	0.00041 (0.001)	−0.001 (0.001)	−0.123 (0.013)***	0.123 (0.013)***
		$E(P_{\mathrm{gj}}\left|\beta_{g}X_{j})\right)$	0.038 (0.002)	0.153 (0.011)	0.063 (0.004)	0.025 (0.004)***	−0.089 (0.012)***	0.115 (0.011)***
	Heterogenity effects	$E(P_{\mathrm{gj}}\left|\beta_{g}X_{j})\right)$–$E(P_{\mathrm{gm}}\left|\beta_{g}X_{m})\right)$	−0.049 (0.005)***	0.097 (0.014)***	0.008 (0.005)*			
		$E(P_{\mathrm{gj}}\left|\beta_{g}X_{j})\right)$–$E(P_{\mathrm{gw}}\left|\beta_{g}X_{w})\right)$	0.036 (0.004)***	0.029 (0.022)*	0.062 (0.006)***			
		$E(P_{\mathrm{gw}}\left|\beta_{g}X_{w})\right)$–$E(P_{\mathrm{gm}}\left|\beta_{g}X_{m})\right)$	−0.086 (0.009)***	0.068 (0.016)***	−0.055 (0.007)***			

Note: g is the sample group where g = m for men manager; g = w for women manager and g = j for joint manager; Figures in parenthesis are standard errors; *, ** and *** indicate statistical significance at 10%, 5% and 1% level.

The average adoption probabilities of ND varieties by men, women and joint plot managers under actual conditions are respectively 51, 34 and 45% in Uganda and 78, 65 and 77% in Tanzania. But, under the counterfactual conditions that the plot managers had had the same characteristics as men managers, the adoption probabilities of ND varieties by women managers compared with men managers would have been lower by 5% in Uganda but higher by 8% in Tanzania. Alternatively, in the counterfactual case that men managers had had the same characteristics as women managers, the adoption probabilities of ND varieties by women managers would have decreased by 21% (0.337 vs 0.547) in Uganda but increased by about 53% (0.652 vs 0.124) in Tanzania, on average, than the probability of ND varieties adoption by men managers. These are the gender gaps in returns between women and men managers.

The adoption gap between men and women managers is different when the gender gap analysis is constructed under different counterfactual conditions. For instance, with the same returns to use of resources as men managers, women’s adoption probability of ND varieties would have increased by about 4% in Uganda (0.547 vs 0.508), and decreased by about 65% in Tanzania (0.124 vs 0.773). These are the heterogeneity or composition effects between men and women managers. Similarly, with the counterfactual condition that men managers’ return had had the women characteristics, women’s adoption probability would have been lower than the adoption status of men managers by 13% in Uganda and 22% in Tanzania. The latter result indicate that there are some important sources of heterogeneity on returns among men and women managers, as well as composition effects that decrease women’s probability of adoption than men under their own observed characteristics.

A similar picture has emerged on the heterogeneity of returns and composition effects between plot managers, with the expected adoption status of DT varieties. We compare men mangers’ average adoption probabilities of DT variety to women and joint plot managers’ adoption status if they had had men managers’ characteristics (counterfactual). In both countries, women plot managers would have less likely to adopt DT than men mangers, however, that the adoption gap would have been higher in Uganda (11%) than in Tanzania (3%). On the other hand, the adoption probability of men managers compared with joint plot managers would be lower by 3% in Uganda and higher by 3% in Tanzania.

The above results merely highlight the adoption gap in returns to resource use between plot managers. But this does not only give an explanation for the overall difference between plot managers with regard to adoption status. We found significant composition effects on DT adoption status among gender of plot managers. The result indicates that in both countries the heterogeneity effects in adoption of DT are higher for women mangers than men mangers. This implies that with the same response levels of resource use as women managers, the adoption of DT by women managers would have increased by about 10% (0.109 vs 0.010) in Uganda and 7% in Tanzania (0.124 vs 0.054). On the other hand, under the condition that when men manger’ coefficients are used, the likelihood of adoption of DT by women compared with men would have increased by about 2% (0.132 vs 0.116) in Uganda but decrease by 9% (0.001 vs 0.086) in Tanzania.

Women and men managers would have decreased adoption of DT had they had, respectively, men’s and women’s resources and characteristics. These differences reflect systematic sources of variation between gender of plot managers that could be fully attributed to differences between women and men in responses as well as to variations in socioeconomic characteristics. In Tanzania, we find asymmetric composition effects on the adoption of DT. In most cases, women are more likely than men to adopt DT varieties. This indicates that there are some important sources of variations on gender returns and composition effects that make women managers more likely to adopt than men managers.

## 5 Conclusions

Technology adoption is a topical issue in the face of changing climate conditions. This research contributes to the existing literature by examining the gender disaggregated adoption of different types of improved maize varieties (such as ND and DT) rather than the adoption of a specific variety alone, which has been the focus of many previous studies. We analyzed the gap between men and women plot managers individually and jointly in their decision as to whether or not to adopt different types of improved maize varieties, modeled by switching regression with multinomial logit model specification.

We found substitution effect on the decision to use a specific maize variety over another variety. This implies that farmers make these decisions jointly and that they are more likely to engage in substitution of one type over the other. The findings of this study have important implications for policy and future research. When DT and ND varieties are substituted for each other, the adoption of a given variety will, in turn, influence the adoption of the other type of variety. Thus, it is important to consider targeted and disaggregated strategies for scaling up the adoption and diffusion of different modern maize varieties, instead of one-size-fits-all approaches.

The result has also shown that with experience in shocks, farmers are responding by increasing the adoption of DT varieties. The result is consistent with the idea that DT is an important option for adapting agricultural production under frequent climatic shock conditions. On the other hand, the bargaining power of men and women, measured by ownership of land control of outputs, may have different effect on the adoption of ND and DT varieties. The effect of gender disaggregated ownership of land on adoption of DT and ND is also differs depending on the gender of the plot manager. It is shown that with the increase in the fraction of area owned by men, adoption of ND is less likely on individually managed plots but more likely on jointly managed plots. On the other hand, with the sole ownership of land by women, the probability of adoption of DT variety is lower on individually managed plots but higher on jointly managed plots. This result would seem to support the collective household model, substantial cooperation between men and women in the households and the role of women’s bargaining power.

The estimation results of the adoption gap between gender of plot managers are different across countries and between the different maize varieties. In Uganda, for instance, the probability of adoption of DT and ND is higher on women managed plots than men managed plots. But plots managed jointly by women and men had shown a higher probability of adoption of both DT and ND varieties than plots managed by men and women individually. In Tanzania, the difference in adoption of DT and ND among gender of plot managers depends on the heterogeneity effects. Some implications have emerged from these results. The results contribute to reshaping our understanding of household decision making and shows that decisions to adopt modern maize varieties cannot be viewed as an isolated decision by the household head but as part of an overall household strategy with different roles and responsibilities of the household members. The decomposition analysis further suggests the difference in returns and composition effects between gender of plot managers for expected adoption of DT and ND varieties. This implies the gender gap (the gap in response to resource use) alone does not give explanation for the overall gender difference in adoption of ND and DT varieties. A combination of policy measures that build individual’s resource capacity and increasing their resources productivity are fundamental to improving the adoption status.

## CRediT authorship contribution statement

**Hailemariam Teklewold:** Conceptualization, Methodology, Formal analysis, Writing - original draft, Writing - review & editing. **Rahma I. Adam:** Conceptualization, Writing - review & editing, Data curation, Supervision. **Paswel Marenya:** Writing - review & editing, Data curation.

## Declaration of Competing Interest

The authors declare that they have no known competing financial interests or personal relationships that could have appeared to influence the work reported in this paper.

## Footnotes

1 We refer plot managers as female head/wife in male-headed households (which we refer as women) and the male head/husband in female-headed households (which we refer as men).

2 The farmer ranked the fertility of each plot as ‘poor’, ‘medium’ or ‘good’, and we derived from that the proportion of farm land with poor and non-poor land.

3 The farmer ranked each plot as ‘flat’, ‘medium’ or ‘steep’ and we derived from that the share of farm land with steep or non-steep slope.

4 For brevity, we did not present the result, but this is available upon request.
